# Oral Squamous Cell Carcinoma Arising in a Patient after Hematopoietic Stem Cell Transplantation with Bisphosphonate-Related Osteonecrosis of the Jaws

**DOI:** 10.1155/2015/831418

**Published:** 2015-04-20

**Authors:** Paolo G. Arduino, Crispian Scully, Luigi Chiusa, Roberto Broccoletti

**Affiliations:** ^1^Department of Surgical Sciences, Oral Medicine Section, CIR Dental School, University of Turin, Via Nizza 230, 10126 Turin, Italy; ^2^WHO Collaborating Centre for Oral Health-General Health, UK; ^3^University College London, Gower Street, London WC1E 6BT, UK; ^4^Azienda Ospedaliera Universitaria Città della Salute e della Scienza di Torino, Corso Bramante 88, 10126 Turin, Italy

## Abstract

A 55-year-old man with a history of acute myeloid leukaemia treated with hematopoietic stem cell transplantation and with a 5-year history of bisphosphonate-related osteonecrosis of the jaws, following 12 cycles of intravenous zoledronic acid therapy, presented in December 2009 with a history of increasingly severe unilateral lower jaw pain. Oral examination revealed, as previously, exposed bone in the left mandible, but also a new exophytic mass on the lower-left buccal mucosa. Biopsy confirmed a diagnosis of oral squamous cell carcinoma. To the best of our knowledge, this is the first report of an oral squamous cell carcinoma that appeared adjacent to an area of osteochemonecrosis.

## 1. Introduction

Bisphosphonate-related osteonecrosis of the jaws (BRONJ) is an important complication mainly reported in patients receiving bisphosphonates (BPs) medication to treat malignant bone metastases and osteoporosis [1].

In 2014, a special committee of the “American Association of Oral and Maxillofacial Surgeons” recommended changing the nomenclature of BRONJ, preferring the term medication-related osteonecrosis of the jaw (MRONJ). The change is justified to accommodate the growing number of osteonecrosis cases, involving the maxilla and mandible, associated with other antiresorptive (denosumab) and antiangiogenic therapies [2].

Although BPs primarily target osteoclasts, soft tissue toxicity has also been reported [3, 4], and there may be some anticarcinogenic effects [5, 6] as well as effects on the immune system [7, 8].

In this report, we describe an oral squamous cell carcinoma (OSCC) that arose adjacent to an area of osteochemonecrosis, related to intravenous BPs therapy.

## 2. Case Presentation

In February 2005, a 50-year-old man was referred for assessment of acute pain in the left posterior mandible of 1 month's duration. The patient had a history of acute myeloid leukaemia treated with hematopoietic stem cell transplantation (HSCT) in 1998. In 2002, he developed multiple bone metastases, for which he received 4 mg of zoledronic acid intravenously over 15 minutes monthly for one year in 2003.

**Figure 1:**
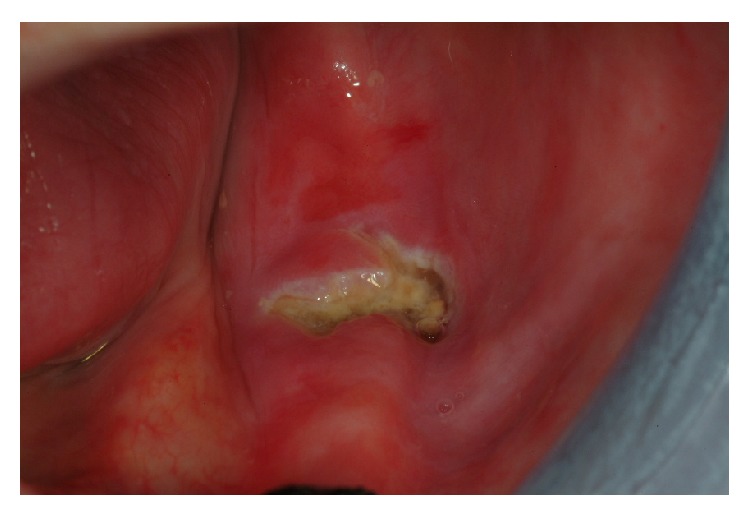
Osteochemonecrosis with exposed necrotic bone on the left mandibular edentulous area.

On clinical examination, there was soreness in the left mandibular area with clinical exposed bone (an area 5 mm in diameter). There was also a purulent discharge and the surrounding gingiva was swollen and erythematous (Figure 1). The patient was totally edentulous, wearing two complete dentures; he did not report any teeth extractions in the previous 4 years.

Dental panoramic radiography and computed axial tomography, and an incisional oral biopsy to rule out a metastasis, confirmed the diagnosis of MRONJ. No other mucosal or osseous lesions were detectable.

Initially, he was treated with antibiotics and chlorhexidine mouth rinse, being however nonresponsive. He declined surgical debridement. He was followed up every 4 months and managed repeatedly with antibiotics [9]. In the follow-up period (October 2007 and February 2008) he developed two new MRONJ lesions in the upper left jaw.

**Figure 2:**
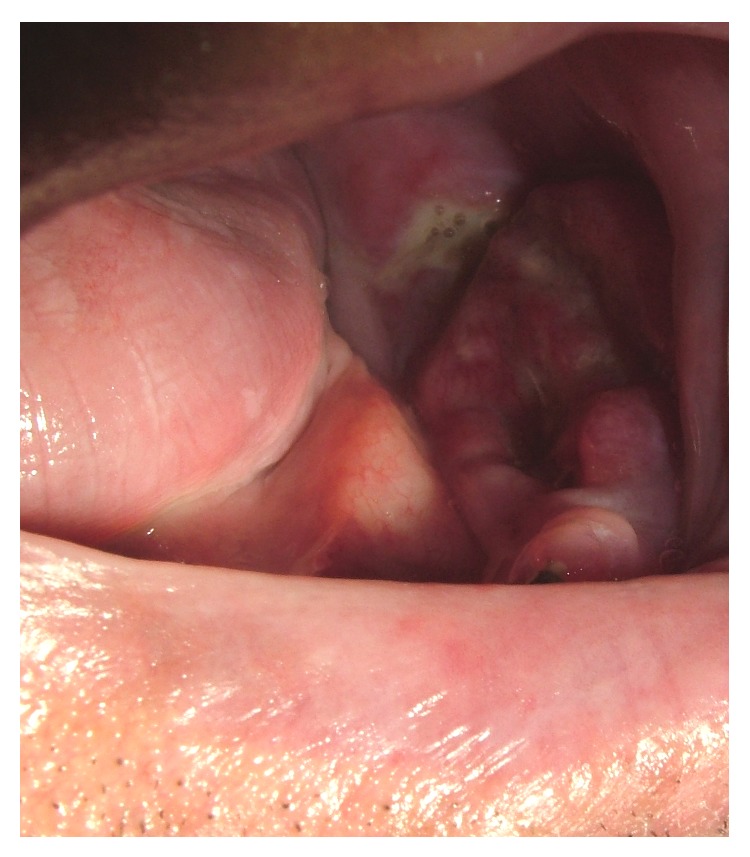
The exophytic mass which developed adjacent to BRONJ, in the left mandible.

**Figure 3:**
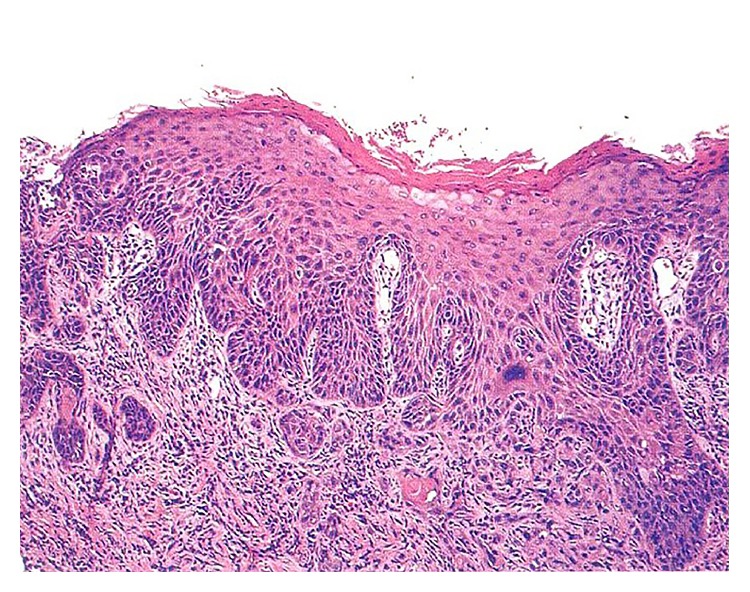
Hematoxylin-eosin-stained section from the proliferative lesion in the left mandibular area, showing invasive squamous cell carcinoma (original magnification 200x).

In December 2009, he presented with a history of increasing severe unilateral lower-left jaw pain. Oral examination revealed, as previously, exposed bone on the left maxilla and a new exophytic mass on the lower-left buccal mucosa (Figure 2). A biopsy of this mass confirmed a diagnosis of OSCC, revealing keratin pearls, nuclear pleomorphism, and connective tissue invasion (Figure 3). He later developed lung metastases and, despite chemoradiotherapy performed, died a year later.

## 3. Discussion

HSCT is broadly used as a potentially curative treatment for patients with various haematological malignancies, bone marrow failure diseases, and congenital immune deficiencies.

To date, despite advances in transplant medicine and in supportive care, oral complications in both autologous and allogeneic HSCT recipients are quite common, including mucositis, infections, oral dryness, taste changes, and graft versus host diseases (GVHD) [10].

In comparison with secondary haematological malignancies, such as posttransplant lymphoproliferative disorders, secondary solid cancers occurring after transplantation are infrequent, but, nevertheless, an increasing number of cases, including OSCC, have been reported [11]. Very recently, it has been reported that recipients of allogeneic HSCT had a significantly higher 2-fold risk of developing secondary solid cancers than the general population [12, 13].

The relative risk of OSCC after hematopoietic stem cell transplantation (HSCT) is increased in males, patients who develop chronic GVHD, patients who have received total body radiation as part of their conditioning regimen, and patients with an over 10-year survival after HSCT [14, 15]. Our patient did not show any of these characteristics, apart from being a male.

Possible pathogenic mechanisms that have been proposed include radiation mutagenesis, GVHD-related inflammation and prolonged GVHD therapy, immunological dysfunction, and carcinogenic and cytotoxic effects of immunosuppressive therapy, or a combination thereof [16].

Above all, it is feasible that malignant oral neoplasms are increasingly being described in the literature as a consequence of lesions of chronic GVHD and prolonged multidrug treatment to control its manifestations (primarily immunosuppressive with azathioprine, cyclosporine, prednisone, or tacrolimus) [16].

Our patient never showed lesion due to oral chronic GVHD; however, his malignancy developed directly adjacent to an area of osteonecrotic bone. We could only presume that the presence of a necrotic infected bone area, in some instance, may have influenced the outcome in a patient partially susceptible.

Oral and dental physicians should therefore be aware that MRONJ lesions might be a possible risk factor for the development of OSCC, particularly in patients with previous HSCT. Further studies to elucidate this possibility are however required.
